# Tracking the Spread of Pollen on Social Media Using Pollen-Related Messages From Twitter: Retrospective Analysis

**DOI:** 10.2196/58309

**Published:** 2024-10-21

**Authors:** Martín Pérez-Pérez, María Fernandez Gonzalez, Francisco Javier Rodriguez-Rajo, Florentino Fdez-Riverola

**Affiliations:** 1 CINBIO, Universidade de Vigo (University of Vigo) Vigo Spain; 2 Department of Computer Science School of Computer Engineering Universidade de Vigo (University of Vigo) Ourense Spain; 3 Next Generation Computer Systems Group, School of Computer Engineering Galicia Sur Health Research Institute, Galician Health Service SERGAS-UVIGO Ourense Spain; 4 Department of Plant Biology and Soil Sciences Faculty of Sciences Universidade de Vigo (University of Vigo) Ourense Spain

**Keywords:** pollen, respiratory allergies, Twitter, large language model, LLM, knowledge reconstruction, text mining

## Abstract

**Background:**

Allergy disorders caused by biological particles, such as the proteins in some airborne pollen grains, are currently considered one of the most common chronic diseases, and European Academy of Allergy and Clinical Immunology forecasts indicate that within 15 years 50% of Europeans will have some kind of allergy as a consequence of urbanization, industrialization, pollution, and climate change.

**Objective:**

The aim of this study was to monitor and analyze the dissemination of information about pollen symptoms from December 2006 to January 2022. By conducting a comprehensive evaluation of public comments and trends on Twitter, the research sought to provide valuable insights into the impact of pollen on sensitive individuals, ultimately enhancing our understanding of how pollen-related information spreads and its implications for public health awareness.

**Methods:**

Using a blend of large language models, dimensionality reduction, unsupervised clustering, and term frequency–inverse document frequency, alongside visual representations such as word clouds and semantic interaction graphs, our study analyzed Twitter data to uncover insights on respiratory allergies. This concise methodology enabled the extraction of significant themes and patterns, offering a deep dive into public knowledge and discussions surrounding respiratory allergies on Twitter.

**Results:**

The months between March and August had the highest volume of messages. The percentage of patient tweets appeared to increase notably during the later years, and there was also a potential increase in the prevalence of symptoms, mainly in the morning hours, indicating a potential rise in pollen allergies and related discussions on social media. While pollen allergy is a global issue, specific sociocultural, political, and economic contexts mean that patients experience symptomatology at a localized level, needing appropriate localized responses.

**Conclusions:**

The interpretation of tweet information represents a valuable tool to take preventive measures to mitigate the impact of pollen allergy on sensitive patients to achieve equity in living conditions and enhance access to health information and services.

## Introduction

### Background

Allergies and associated diseases such as asthma, rhinosinusitis, atopic dermatitis, and food-oral syndrome affect at least 30% of the world population and close to 80% of families [1]. The World Health Organization estimates that 400 million people in the world experience allergic rhinitis and 300 million experience asthma [2]. In particular, allergy disorders caused by biological particles as proteins of some airborne pollen grains are currently considered one of the most common chronic diseases. European Academy of Allergy and Clinical Immunology forecasts indicate that, within 15 years, 50% of Europeans will have some kind of allergy as a consequence of urbanization, industrialization, pollution, and climate change [3-8]. Therefore, in less than half a century, allergy, originally perceived as a rare disease, has become a major public health threat.

According to recent data published by the European Academy of Allergy and Clinical Immunology, allergy constitutes a significant cause of morbidity worldwide, considered a pandemic with an impact on the quality of life and the health and medical systems of both resource-rich and resource-limited economies [9,10]. An important impact of pollen levels is observed in the increase in overconsultations in primary care for allergic rhinitis [11]. Moreover, it is estimated that, in Europe, asthma and rhinitis caused by allergies generates >100 million days of absenteeism at work and schools, in addition to the economic losses due to presenteeism, a circumstance in which people go to work but their productivity is extremely reduced [3]. Some studies estimate that the total cost of these allergic disease–induced situations to the United States was US $19.7 billion, and in the European Union, economic losses ranged from €55 to €151 billion (US $61 to US $167.5 billion) [4,12].

Preventive measures during high pollen seasons are medications; immunotherapy; and the adoption of strategies such as staying indoors during peak pollen times, using air purifiers, and keeping windows closed to reduce exposure [13]. The most common information available to prevent allergies for patients with pollinosis is the number of pollen grains or aeroallergens in the bioaerosol and their temporal distribution [10,14].

In addition, the timing of symptomatology of allergenic people represents suitable information to prevent allergies, but the difficulty of real-time patient symptom monitoring makes it very hard to obtain this kind of data. To solve this gap, epidemiological studies could be conducted with new technological tools such as the assessment of social media data to track the spread of pollen symptomatology–related messages [15,16]. During recent years, social networks have also become a significant tool for business and science dissemination and a reference to understand and study the trends, ideas, feelings, and thoughts of individual users and, therefore, are turning into a great opportunity to carry out new methodological studies in which individual people’s accounts participate as coproducers of scientific knowledge [15,17,18]. Patient narratives on social networks can provide valuable insights into public awareness and seasonal patterns to take preventive measures to mitigate the impact of pollen on sensitive patients. A better understanding of exposure and symptom levels will help significantly improve new therapies [19].

Several key factors should be considered for an accurate social media data analysis. The accurate identification of platforms where users frequently discuss health-related topics, such as Twitter (subsequently rebranded X), Facebook, Instagram, and health forums, is essential to selecting relevant keywords related to pollen, allergies, or related symptoms [20,21]. Among all the currently existing social networks, Twitter has a leading role as a broadcasting communication medium for researchers, stakeholders, or organizations [15,16,22-25]. Twitter has been used to extract public opinions and attitudes in previous health studies related to COVID-19 [26] or Alzheimer disease [27], demonstrating how Twitter discourse analysis can provide insights into public opinions and attitudes on health and social issues. In addition, it is important to define the precise period for an accurate social media retrospective analysis considering seasonal variations in pollen levels. For accurate and current insights from global social media analysis, (1) irrelevant content such as retweets, spam, and duplicates must be meticulously removed during data cleaning [15,22]; (2) the frequency of pollen-related messages over time to identify peak periods should be determined; and (3) the geographical distribution of pollen-related discussions should be explored to identify regions with earlier prevalence, which serve as forecast for other regions, and trends in pollen-related discussions [17]. Comparing social media data with official pollen count data reveals valuable insights—online discussions correlate with actual pollen levels, pinpointing periods of peak pollen-related messages that suggest high pollen activity. This information can inform recommendations for health organizations, policy makers, and the public, tailored to address health trends related to different conditions [15,21,24].

### Objectives

In this context, this study aimed to leverage advancements in data analysis and social networks using the semantic knowledge embedded in the latent space of large language models (LLMs), unsupervised clustering, term frequency–inverse document frequency (TF-IDF), and graph knowledge reconstruction techniques to track and scrutinize the dissemination of messages related to pollen symptoms from December 2006 to January 2022. This sophisticated approach facilitated a detailed examination of public discussions and trends on Twitter, offering insights into the impact of pollen on sensitive individuals.

## Methods

### Overview

**Figure 1:**
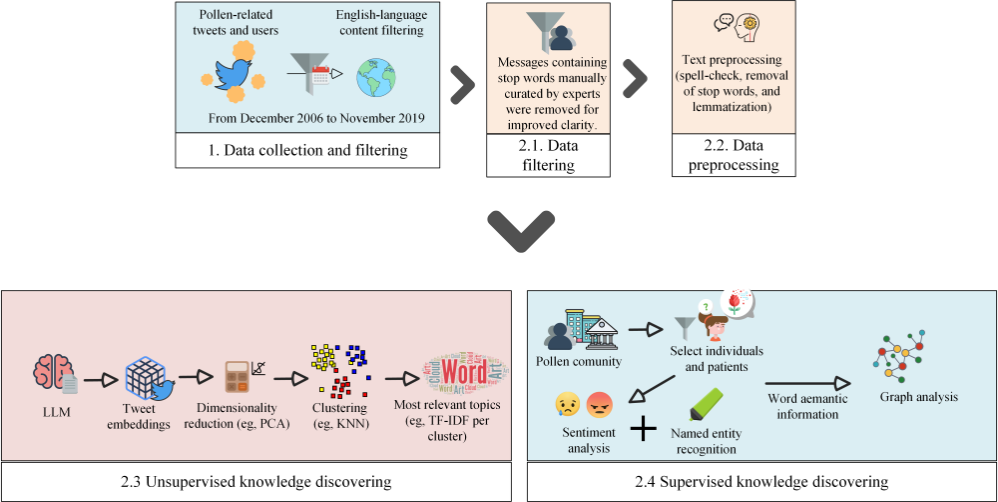
Applied analysis workflow. The analysis began with collecting pollen-related tweets and user data, filtered for English content and curated for problem domain relevancy. KNN: k-nearest neighbor; LLM: large language model; PCA: principal component analysis; TF-IDF: term frequency–inverse document frequency.

This section summarizes the different steps and methods applied to obtain pollen-related knowledge on the Twitter social network. Figure 1 presents the workflow applied following the methods described in the following sections.

This analysis focused on pollen-related tweets and user data collected from December 2006 to January 2022. Tweets were filtered to retain only English-language entries and remove those irrelevant to the study domain using a handcrafted stop word list (eg, spam messages or nonrelevant topics). The refined data then underwent text preprocessing, including spell-checking, standard stop word removal, and lemmatization to normalize the word representation.

Upon completing the text preprocessing, both supervised and unsupervised methods were applied. In the unsupervised workflow, the messages were encoded using LLMs to map out semantic latent spaces as word embeddings. These embeddings were then dimensionally reduced via principal component analysis (PCA) to simplify the dataset complexity while preserving its essential structure. Subsequently, clustering algorithms such as k-nearest neighbor (KNN) were used to identify natural groupings within the data. To pinpoint the most pertinent topics within each cluster, further analysis applied the TF-IDF method, which highlights words that are more significant within a cluster relative to the entire dataset.

On the other hand, in the supervised workflow, usernames and descriptions were automatically analyzed to classify individual and nonindividual profiles (eg, public entities or news channels). Sentiment analysis was performed to assign emotional values to tweets and gauge public sentiment, whereas named entity recognition (NER) was applied to identify and categorize key entities relevant to the problem domain, such as symptoms or diseases. Finally, graph analysis was conducted to visualize the semantic interactions of the annotated words. This comprehensive approach provided a detailed understanding of the discourse and patterns related to pollen and its effects on the community throughout the analyzed period.

The following sections provide a more detailed exploration of the methods implemented in each component of the workflow described previously.

### Data Collection and Filtering

To carry out the proposed analysis, a collection of 4.6 million English-language tweets that mentioned the word *pollen* from December 2006 to January 2022 were collected using the Twitter4J library, a Java tool for the Twitter application programming interface. To obtain relevant information in the topic domain, some repeated tweets and tweets mentioning noninteresting words were filtered out during the data collection process as well as retweets to prevent redundant information. However, it is important to note that not all collected messages contained relevant biomedical knowledge. The term *pollen* is used by the Twitter community to refer to a variety of topics, including video games, digital coins, insects, photography, and pollen level alerts. To ensure the relevance and specificity of the dataset, the tweets were meticulously filtered to exclude messages that were not relevant to the domain of the study. This semiautomatic filtering process involved retaining only English-language tweets and removing those with content irrelevant to the research focus. A carefully curated stop word list developed by a domain expert was used to exclude messages that were deemed unconnected to the health-related research topic. This list included words associated with unrelated subjects, such as *bees*, *weather channels*, and *pollen products for sale*, among others. This data curation approach helped refine the whole dataset, ensuring that the analysis was focused and relevant to the health domain.

### Data Preprocessing

The content of the collected tweets could vary from useful and meaningful information to unconnected text. To extract high-quality information and features, certain preprocessing techniques were required. Therefore, the following preprocessing steps were applied.

Different techniques were applied to normalize the user messages, namely, (1) URL identification and filtering, (2) word normalization (eg, *haaaaaaaaaaaaappy* to *happy*), (3) removal of extra white space, and (4) spelling error correction using the Hunspell dictionary with the normalized Levenshtein algorithm [28]. Finally, the mentions of Twitter users were identified and filtered to ensure compliance with privacy and ethical considerations.

Different techniques were applied to preprocess texts for topic discovery, clustering, and NER. The semantic normalization processing included tokenization to separate the text into meaningful elements such as words and phrases; removal of common and low-value words such as English stop words or domain-specific ones (eg, pollen); categorization each token using part-of-speech tagging; filtering of numbers and tokens with <2 characters; transformation of all tokens to lower case; and, finally, standardization of tokens to use only their root form through lemmatization techniques.

### Extracting the Main Discussed Topics in Social Media Conversations

To uncover the main topics discussed by the community within the evaluated time frame (December 2006-January 2022), this study implemented (1) a topic modeling strategy using large language embeddings, specifically, Bidirectional Encoder Representations From Transformers (BERT), to explore the semantic latent space; (2) a vector dimension reduction (ie, PCA); (3) clustering (ie, KNN); and (4) a word relevance statistical analysis method (TF-IDF) to discover dense clusters (eg, group-discussed subjects) and easily describe and interpret the most relevant discussed topics. The following sections describe each processing technique.

#### Applying LLM Semantic Latent Space to Discover the Main Social Media Topics

Document embeddings are dense vector representations that capture the semantic latent space, enabling mathematical comparisons of text based on the vector space similarity. By representing documents as real-valued feature vectors, it becomes possible to perform topic modeling and other natural language processing (NLP) tasks, measuring the closeness of documents in vector space. BERT, one of the most widely used language representation models, has established itself as a benchmark for NLP tasks due to its ability to extract high-quality embeddings from text corpora [29,30]. The BERT model has been widely used for text classification tasks. To build its understanding of language, BERT pretrains a deep neural network on a large corpus of text data. This approach gives the model a comprehensive understanding of the semantic structure and context of language. BERT’s bidirectional nature enables it to interpret the context of words by analyzing those that precede and follow each word in a sentence, leading to superior performance compared to traditional language models. BERT has been successfully applied to various text classification tasks, including sentiment analysis, topic classification, and fake news detection, often producing state-of-the-art results. BERT models can also be used for clustering tasks by leveraging their semantic latent space. BERT generates high-dimensional embeddings that capture the contextual and semantic information of the input text. In this sense, BERT embeddings were used to extract text features and group similar social media discussions together via clustering algorithms such as k-means clustering.

#### Dimensionality Reduction

The high-dimensional nature of LLMs such as the BERT model generates embeddings with hundreds or thousands of dimensions multiplied by the number of messages to analyze, which can be computationally expensive and memory intensive to work with. In addition, the large extension of the dataset generated from social media data makes it even more expensive to process the data.

In this context, dimensionality reduction techniques such as PCA are emerging as effective tools when combined with LLMs to decrease dataset dimensions. This integration simplifies data handling and enhances its visualization and interpretability [31]. PCA is a linear technique that identifies the directions in the data that capture the most variation and projects the data onto a lower-dimensional space along these directions. In this context, PCA was used to condense the semantic vector space matrix, thereby optimizing both performance and the representation of the evaluation steps.

#### Clustering Topic Analysis

Clustering methods such as KNN are fundamental techniques in the field of text mining. KNN is a straightforward and effective strategy for grouping similar data points based on their proximity to each other. This method facilitates the discovery of unsupervised knowledge by bringing together similar text documents, thereby revealing patterns and connections within the data that might not have been immediately clear. The integration of KNN with BERT latent space offers a powerful methodology for unsupervised text analysis. When texts are transformed into embeddings, the resulting vectors contain a vast amount of semantic information. This allows for the comparison of texts and the measurement of similarity based on these semantic features. In the context of clustering, these embeddings can serve as input for KNN, enabling it to identify and group texts based on their semantic similarities. In this way, a text can be assigned to the cluster of the k-nearest texts considering the similarities in their embeddings. This approach helps in categorizing text documents efficiently and effectively, illuminating hidden structures within large sets of data. When these embeddings are processed using KNN, the algorithm can accurately group tweets with similar content, thereby identifying prevailing topics within the Twitter discourse.

#### Word Relevance and Clustering Representation

TF-IDF is a common representation used in text analysis for the importance of a word in a document in relation to a corpus of documents. TF-IDF is calculated as the product of 2 measures: the term frequency and the inverse document frequency. The term frequency measures the frequency of a word in a document, whereas the inverse document frequency measures the rarity of a word in the dataset. In the context of clustering, the TF-IDF representation was used to identify the keywords or phrases that are characteristic of a particular cluster and to provide a more interpretable and concise representation of how the social community expresses itself and associates every concept or eventual idea. TF-IDF is defined as follows (equation 1):

*tf* – *idf*(*t*, *s*, *D*) = *tf*(*t*, *s*) × *idf*(*t*, *D*),

where *t* is the evaluated term, *s* stands for any given sentence of the dataset *D*, and *tf*(*t*, *s*) expresses the ratio corresponding to the term *t* in a sentence *s*, described as follows (equation 2):



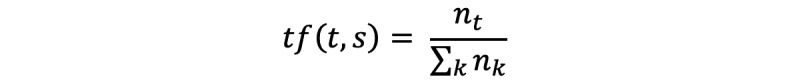



where *n_t_* is the number of occurrences of the term *t* in a sentence *s* and *n_k_* is the total number of terms in a sentence *s*. Moreover, in equation 1, *idf*(*t*, *D*) stands for the logarithmic ratio of the term *t* in the dataset *D* and is computed as follows (equation 3):



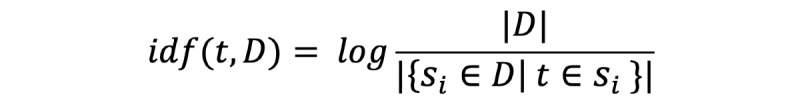



### Extracting Health-Related Patterns in Social Media Discussions

#### Overview

Analytical methods, including NER and coattention semantic networks, complement broader analytical efforts by extracting key insights from biomedical and health-related datasets. These techniques leverage labeled data and predefined categories to pinpoint crucial information effectively. By integrating the outcomes of this guided research with sentiment and semantic graph analyses, it is feasible to identify novel patterns and connections within the data that might otherwise remain elusive. Therefore, to discover relevant knowledge, the following techniques were applied.

#### NER Technique

NER enables a more accurate and comprehensive health-related analysis of medical topics discussed on social media compared to unsupervised methods that only rely on usual word frequency and patterns. By leveraging NER, relevant medical information can be extracted and analyzed, uncovering important insights and knowledge that might otherwise go unnoticed in the studied domain. Therefore, in terms of term recognition, an automatic processing workflow supported by different automatic state-of-the-art recognizer tools was established to identify relevant health topics in the problem domain.

For this process, the following 6 state-of-the-art NER taggers were used to assist the annotator and save efforts in the semiautomatic annotation workflow: TMCHEM [32] to identify chemical, drug brand, and trade names; LINNAEUS [33] to annotate species; disease name normalization (DNORM) [34] to recognize disease names; A Biomedical Named Entity Recognizer (ABNER) [35] to annotate genes and proteins; and Open Source Chemistry Analysis Routines (OSCAR4) [36] to recognize chemical names, reaction names, enzymes, chemical prefixes, and adjectives. In addition, to complement the annotation process and annotate the domain categories of *Disease*, *Food & Nutrition*, *Anatomy*, *Drug and Chemical compounds*, *Symptoms*, *Plants*, and *Diet*, an in-house ontology-based NER was established based on the following ontologies: the FoodOn ontology [37], the National Cancer Institute Thesaurus ontology [38], the Symptom Ontology [39], the Foundational Model of Anatomy ontology [40], the Medical Subject Headings ontology [41], the Chemical Entities of Biological Interest lexicon [42], the Disease Ontology [43], the Experimental Factor Ontology [44], the International Classification of Diseases [45], the Interlinking Ontology for Biological Concepts [46], the Human Phenotype Ontology [47], the DrugBank lexicon [48], the Kyoto Encyclopedia of Genes and Genomes lexicon [49], and a list of domain terms manually curated by experts.

This in-house ontology-based NER entailed a dictionary lookup as well as pattern and rule-based annotation to perform an inverted recognition strategy in which sentence words were used as patterns to be matched against the established lexicon from the ontologies and the manually curated list of domain terms [50]. In this way, the lexical knowledge provided by the different ontologies allowed for the recognition of perfect matches as well as lexical variations of the terms (ie, lemmatized entries and synonyms).

#### Coattention Semantic Graph Reconstruction

Coattention semantic graphs represent a valuable asset for extracting pertinent insights into the dynamics of how social media communities engage with different health-related subjects. This approach enables a more nuanced understanding of public sentiment, prevalent misconceptions, and emerging concerns, thereby offering a comprehensive view of the collective health discourse on social platforms. These graphs are built by analyzing the frequency and patterns of co-occurrence of terms in a corpus of text data, such as social media posts or online articles. The resulting graph represents a map of the semantic relationships between the different health-related concepts discussed, with nodes representing the word concepts (eg, *Itchy eye*, *Runny nose*, or *fatigue*), edges representing the existence of a discussed relationship in the community, and the weight of each edge representing the strength of the co-occurrence relationship between them (ie, the number of social media messages that discuss both terms in conjunction). Therefore, the concepts identified through NER were used to reconstruct co-occurrence semantic graphs of the Twitter messages that were evaluated. First, the relevant terms were identified using NER, which were then analyzed for their frequency of co-occurrence. The knowledge graph was then reconstructed by connecting the identified entities based on their semantic comention. This process enables the discovery of hidden relationships and patterns in the data, providing valuable insights into the health-related relationships between different biomedical entities identified in the text data. This technique serves as a valuable methodology to analyze the degree of association between various health topics (eg, disease, symptoms, or treatments) discussed by a specific social media community (eg, patients or users expressing themselves in the first person).

#### Sentiment Analysis

Different studies have used sentiment analysis to explore the emotional component of tweets. Using NLP techniques, these analyses assessed the words or symbols in tweets to quantify the intensity of positive and negative sentiments or emotions [51,52]. Therefore, and to gain insights into the sentiment of the messages posted on the social network concerning the main topic, sentiment analysis was carried out using the pretrained model Twitter-Robustly Optimized BERT Pretraining Approach-base for Sentiment Analysis [53]. This model was trained on a vast dataset of 58 million tweets and fine-tuned for sentiment analysis using the TweetEval benchmark, making it an effective tool for analyzing sentiment in social media messages.

### Graph Analysis

To visualize the results of semantic graphs, the Gephi tool [54] and the Circle Pack layout [55] were used. Gephi is a widely used open-source network analysis and visualization tool that allows for the exploration and interpretation of complex graphs. The Circle Pack layout is a hierarchical layout algorithm that arranges nodes in a circular packing order, with the most important nodes usually positioned in the center and the less important ones in the periphery. In this way, the Circle Pack layout can help identify the most central and significant entities in the graph, making it an effective way to visualize the results.

Finally, to gain a deeper insight into the structure and the embedded knowledge, various graph statistical metrics were computed to describe their characteristics. State-of-the-art metrics such as degree, betweenness centrality, closeness centrality, characteristic path length, clustering coefficient, and the average number of neighbors were used for this purpose [56-58]. Degree centrality quantifies the total number of direct links (eg, the number of relations) to other vertices, and higher degrees indicate greater centrality. Betweenness centrality measures a node's importance in facilitating communication between other nodes. A node with higher betweenness centrality plays a more critical role, as many paths between other nodes must pass through it. Finally, closeness centrality measures the ease of connectivity between vertices, with a high closeness centrality corresponding to nodes with short average shortest paths to other nodes (eg, the ease with which a term is mentioned by the community) [59,60].

### Community Characterization

The user profiles in this study underwent data collection and processing to classify the accounts involved in the conversations. Consequently, to save manual processing efforts and improve the final results, an automatic user characterization workflow was established based on the previous works of the authors in the problem domain [61]. The purpose of this step was to discern between individual and nonindividual users (ie, organizations; stakeholders; and educational, informative, or commercial accounts without personal connections), determine their sex (male or female), and identify their association with the main topic (ie, accounts belonging to patients with mentioned pollen allergy). This user-centered analysis allowed for the stratification of the final outcomes in terms of health.

### Ethical Considerations

All data were obtained from the public social media platform Twitter using their application programming interface. In addition, the information obtained was freely published by the different users of the platform and remains under their control, allowing them to delete it at any time. The results generated do not contain any user-identifiable data, ensuring the privacy and anonymity of the individuals involved. Our methodology emphasizes aggregate trends and patterns rather than individual user behaviors.

## Results

### Knowledge Base Description

As previously mentioned, all user messages were preprocessed to discern relevant and nonrelevant messages to the health topic. In this sense, a total of 4.6 million messages were processed to create a final evaluable knowledge base of relevant tweets. The final dataset consisted of 4,643,994 tweets after the preprocessing and filtering steps. Of these, 1,393,304 tweets (30%) were deemed relevant to health, while 3,188,607 (68.7%) tweets were considered irrelevant. The preprocessing filtering step ensures a clearer understanding and deeper insights into the topics of interest, improving the final results and making the data more useful for discovering knowledge and decision-making. To carry out this process, a subsample of tweets was revised by an expert to select the most common words that were not related to the problem domain. Then, a fully automatic process filtered all tweets, or user accounts, that contained these words. Textbox 1 represents an extract of the nonrelevant topics of the filtered messages, some of them related to weather accounts and bee, honey, or pollen seller messages.

**Nonrelevant words and bigrams of the filtered messages. The textbox illustrates an extract of the top 15 nonrelevant topics from the filtered messages scaled by their occurrence in the knowledge base.**

**Top 15 nonrelevant words**

- Bee
- Health
- Honey
- Plant
- Grain
- Benefit
- Fenner
- Food
- Discover
- Try
- Use
- Superfood
- Purifier
- Profile
- Eat

**Top 15 nonrelevant bigrams**

- Bee-honey
- Bee-health
- Benefit- health
- Fever-hay
- Rain-wash
- Discover-health
- Profile-superfood
- Discover-profile
- Count-mold
- Grass-weed
- Contain-preservative
- Plant-release
- Health-increase
- Color-preservative
- Plant ragweed

**Figure 2:**
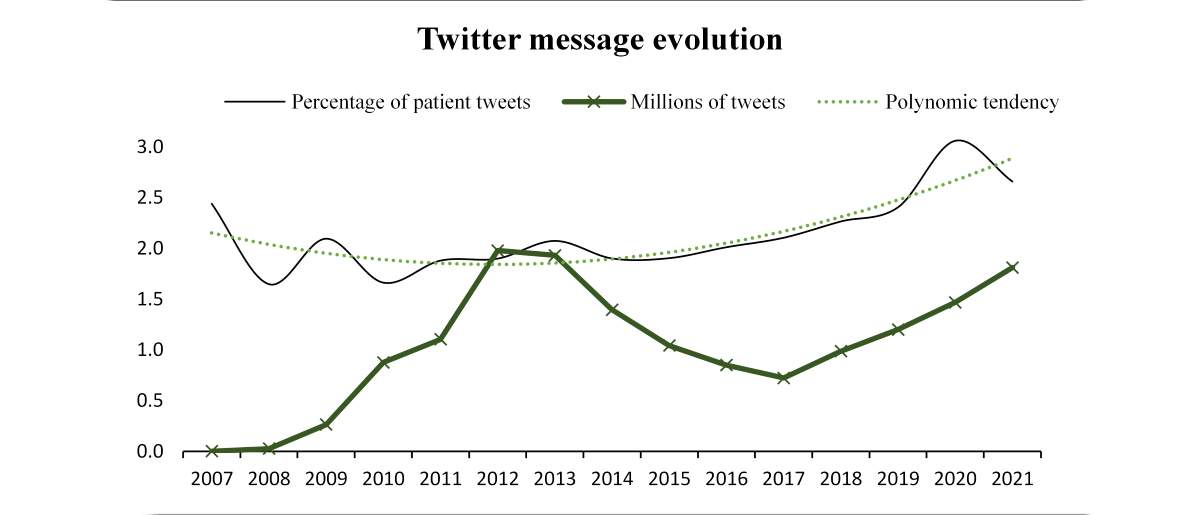
Evolution of "pollen" discussions on Twitter from December 2006 to January 2022. The figure shows the annual percentage of tweets mentioning “declared patients” affected by pollen allergies, with a polynomial trend line indicating overall tendencies.

To gain insights into the progression of the dataset over time, Figure 2 shows the evolution of discussions on social media related to the *pollen* topic. The figure shows the percentage of tweets mentioning “declared patients” affected by pollen allergies (y-axis) each year (x-axis). This percentage was calculated by dividing the number of tweets mentioning patients who were affected by pollen allergies by the total number of tweets in that year. The graph includes the overall trend in the total number of tweets and a polynomial trend line, indicating the general tendency over the years. This graphical approach not only provides insights into the frequency of conversations but also enables analysis of the volume of discussions over time. Therefore, as can be observed in Figure 2, the years in which the word *pollen* was used in the greatest volume worldwide (in the context of health) were 2012 to 2013 and 2020 to 2021.

Along the same line, and to visually analyze the trends in patient engagement with the topic of *pollen* over time and provide insights into the changing levels of interest and engagement among patients, Figure 2 also shows the trends in patient tweets as a proportion of the total number of tweets for each year from December 2006 to January 2022 (ie, patients who mentioned their allergic illness over time). The data show a clear upward trend, with a peak of 3.06% (4493/146,825) of patient tweets in 2020. In particular, the percentage of patient tweets appeared to increase notably from 2018 to 2020, indicating a potential rise in pollen allergies and related discussions on social media. On the other hand, and with a deep granularity, the data suggest that there is a clear and expected seasonal pattern in the discussion of pollen on Twitter. The months between March and August had the highest volume of messages. April was the month in which the community discussed pollen the most. These data were in accordance with those of another health-related social media study conducted in the United States that pointed out that most tweets per month associated with telehealth for mental health or substance abuse were posted from March 2020 to June 2021, with a peak in May 2020 [62].

Accordingly, for the whole period under analysis, the days between May 7, 2018, and May 10, 2018, correspond to the period of the greatest popularity of the word *pollen* on Twitter (also in terms of favorites and retweets). In terms of year of popularity, 2021 was the year with the highest number of retweets and favorites concerning the topic, which demonstrates a growing interest in pollen and a growing information flow.

To identify the most discussed subjects in the community, an unsupervised topic analysis of the relevant messages (1.39 million) was carried out. Therefore, Table 1 represents the top 12 main topics discussed from December 2006 to January 2022. To obtain the most important words for each topic, the TF-IDF word relevance technique was used. The x-axis represents the TF-IDF word relevance of each word or term (ranging from 0 to 1) within the set of evaluated messages. The results obtained showed that the principal symptoms mentioned were related to ailments such as eye problems, allergic reactions, hay fever, and other symptoms (nose and head related). The data illustrate that conversations not only were centered on physical symptoms such as eye irritation, sneezing, and respiratory issues but also encompassed a strong emotional and social component.

The presence of strong language and expletives reflects elevated levels of frustration and discomfort among individuals affected by pollen allergies. Moreover, the association of pollen with weather-related terms such as *rain*, *wind*, and *mold* suggests that public awareness and concerns about pollen allergies are closely linked to environmental conditions. This could indicate that people are turning to social media to seek advice or share individual experiences related to weather changes affecting their allergy symptoms. One key finding was the significant mention of specific environments and activities, such as *street*, *night*, *morning*, and *work*, suggesting that discussions about pollen allergies are often contextualized within everyday scenarios and concerns. Furthermore, the prominence of terms related to emotional responses and coping strategies, such as *happy*, *hate*, *fight*, and *help*, underscores the emotional burden of pollen allergies and the community’s efforts to seek and offer support. Finally, the analysis revealed a nuanced conversation about weather and seasonal changes, indicated by the association with terms such as *weather*, *wind*, *rain*, and climatic change This suggests that public concerns and experiences with pollen allergies are closely tied to weather patterns and climate change phenomena. The same trend has been pointed out by other authors who measured the frequency of the words *allergy*, *asthma*, and *rhinitis* in tweets identifying the weather as the most relevant topic related to *asthma* and *rhinitis* [63].

**Figure 3:**
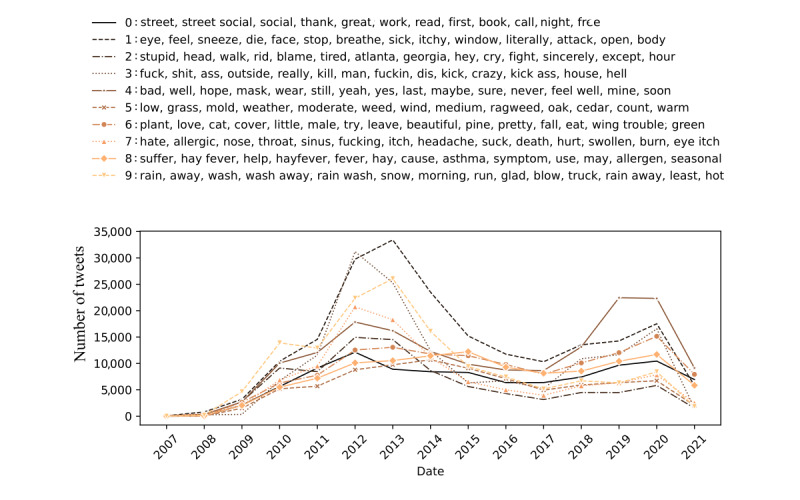
Evolution of the different topic clusters in the pollen-related discussions and their relevance from December 2006 to January 2022.

In addition, a temporal analysis of the identified topic clusters was carried out to obtain insights into the dynamics of online discussions and the evolution of public opinion. Therefore, the temporal analysis of social media data can reveal historical trends and the relationships between different topics, providing a comprehensive view of the dynamics of online discussions and their evolution over time. Figure 3 illustrates the progression of the main topics identified over the assessed period. The graph shows the relevance of each cluster over time, illustrating the fluctuating interest and discussion trends within the Twitter community on pollen-related topics.

**Table 1:** Top 12 main topic clusters showing the different concepts discussed together from December 2006 to January 2022.

		TF-IDF^a^
**Topic 0**		
	Street	0.22
	Street social	0.21
	Social	0.21
	Thank	0.16
	Great	0.13
	Work	0.12
**Topic 1**		
	Eye	0.38
	Feel	0.29
	Sneeze	0.26
	Die	0.10
	Face	0.09
	Stop	0.09
	Breathe	0.08
**Topic 2**		
	Stupid	0.09
	Head	0.07
	Walk	0.05
	Rid	0.04
	Blame	0.04
	Tired	0.03
	Atlanta	0.03
**Topic 3**		
	Fuck	0.36
	Shit	0.31
	Ass	0.28
	Outside	0.25
	Really	0.18
	Kill	0.17
	Man	0.14
**Topic 4**		
	Bad	0.33
	Well	0.23
	Hope	0.14
	Mask	0.14
	Wear	0.10
	Still	0.09
	Year	0.09
**Topic 5**		
	Low	0.36
	Grass	0.35
	Mold	0.25
	Weather	0.23
	Moderate	0.22
	Weed	0.15
	Wind	0.14
**Topic 6**		
	Plant	0.31
	Love	0.22
	Cat	0.11
	Cover	0.11
	Little	0.11
	Male	0.10
	Try	0.09
**Topic 7**		
	Hate	0.28
	Allergic	0.28
	Nose	0.22
	Throat	0.10
	Sinus	0.09
	Fucking	0.07
	Itch	0.06
**Topic 8**		
	Suffer	0.23
	Hay fever	0.19
	Help	0.19
	Hayfever	0.19
	Fever	0.19
	Hay	0.18
	Cause	0.15
**Topic 9**		
	Rain	0.56
	Away	0.33
	Wash	0.32
	Wash away	0.25
	Rain wash	0.17
	Snow	0.10
	Morning	0.09

^a^TF-IDF: term frequency–inverse document frequency.

The consistent pattern observed across the data series presented in Figure 3 hints at a close connection between the different topics identified, suggesting that some years had a greater impact on the community (2011-2014). While most topics (topics 1, 2, 3, 7, and 8) primarily addressed medical concerns, expressions about health status, and symptomatology associated with pollen allergies, there were distinct topics that diverged to explore various other subjects. This variety in subject matter reflects the multifaceted nature of public discourse surrounding pollen allergies, encompassing not only health-related issues and community support but also broader environmental, social-cultural, and external events that have influenced the community. For example, topic 6 provides a broad perspective on pollen, addressing its presence in various contexts from environmental exposure to its role in natural beauty and even as a food supplement. The interplay of terms such as *plant*, *beautiful*, and *eat* suggests an acknowledgment of pollen’s complexity, encompassing its allure and nutritional aspects. Since 2019, the discourse on mask use (topic 4) shifted significantly due to the COVID-19 pandemic, impacting discussions related to pollen allergies. This shift would be particularly visible in temporal data, where an uptick in mask-related discussions could coincide with the pandemic’s onset, reflecting a broader societal adaptation to mask wearing.

### Male Versus Female Topic Analysis

To better understand the primary conditions discussed in relation to accounts classified as male and female, the methodology described in the Extract Main Discussed Topics in Social Media Conversations section was applied to discover the main mentioned topics by sex. This approach specifically targeted messages from accounts identified by their sex, allowing for a detailed analysis of the health topics predominantly associated with each group. This sex classification was determined based on the demographic insights derived from the authors’ previous research in the problem domain [61]. The messages attributed to each sex was segregated into 2 distinct clusters. Within these clusters, the top 7 topics demonstrating the highest impact, as measured using their TF-IDF scores, were determined. Table 2 represents the key topics discussed in relation to male- and female-classified accounts and measured by their term TF-IDF word relevance within the evaluated messages. Although no substantially differences were observed overall, it was found that asthma and sneezing were more significant discussed topics in the female messages.

**Table 2:** Most common pollen-related topics discussed by male and female individuals, and measured by their term frequency–inverse document frequency (TF-IDF).

				TF-IDF
**Top male-discussed topics**				
	**Topic 0**			
		Hayfever	0.03	
		Sinus	0.03	
		Eye	0.03	
		Rain	0.02	
		Nose	0.02	
		Grass	0.02	
		Kill	0.02	
	**Topic 1**			
		Big snort	0.69	
		Snort sinus	0.68	
		Sinus big	0.63	
		Snort	0.39	
		Big	0.39	
		Sinus	0.25	
		Dammit sinus	0.05	
**Top female-discussed topics**				
	**Topic 0**			
		Eye	0.03	
		Hayfever	0.03	
		Sinus	0.02	
		Sneeze	0.02	
		Asthma	0.02	
		Rain	0.02	
		Nose	0.02	
	**Topic 1**			
		Big snort	0.88	
		Snort sinus	0.83	
		Sinus big	0.72	
		Big	0.43	
		Snort	0.40	
		Sinus	0.37	
		Pretty sure	0.06	

### Semantic Connections of Medical Topics

**Figure 4:**
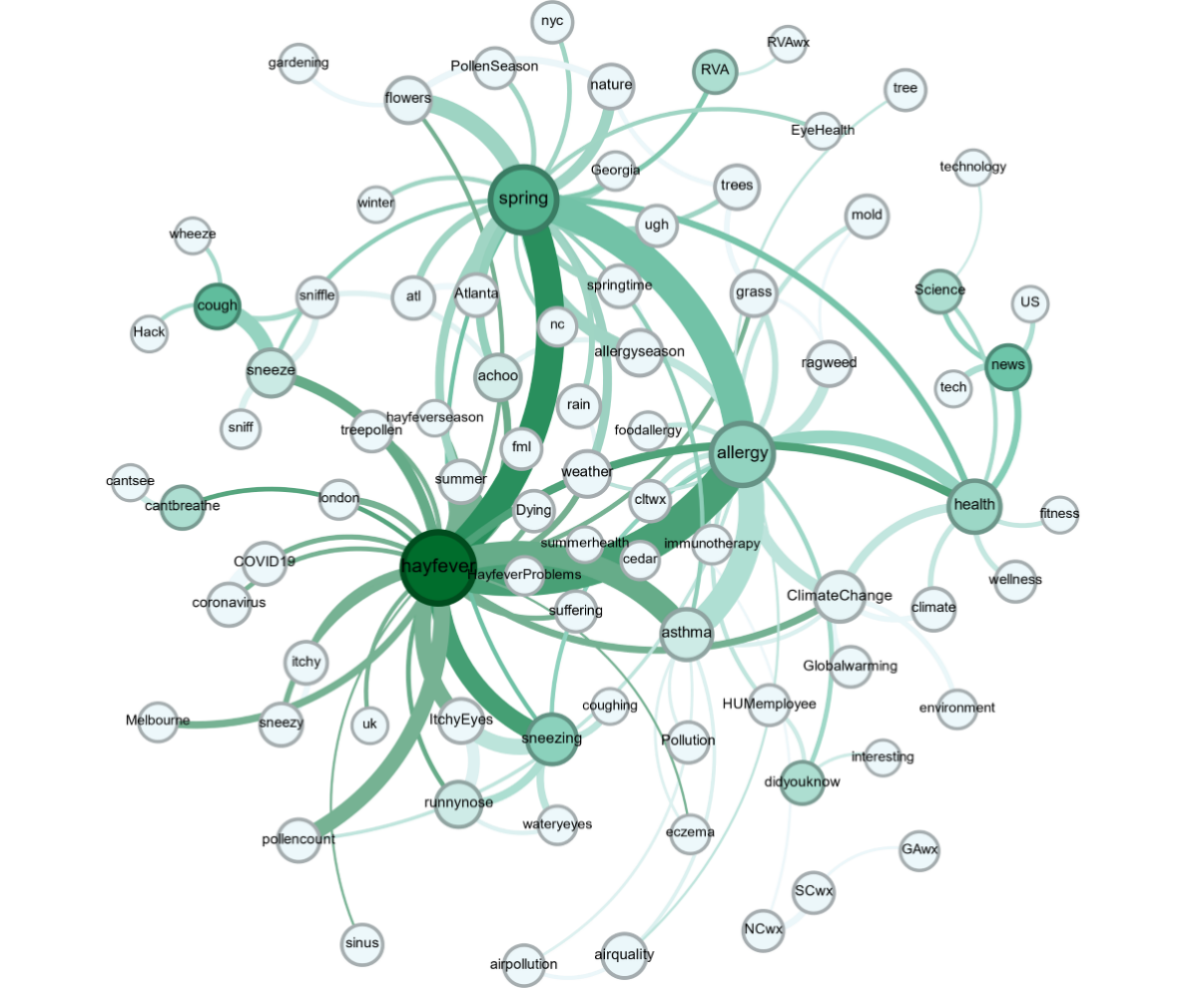
Top comentioned hashtags in pollen-related discussions. Knowledge graph representing the comention hashtags from December 2006 to January 2022.

Different semantic knowledge networks were reconstructed to offer valuable insights into the behavior and semantics that the community uses to discuss. This approach has been used by other researchers as a method to uncover new knowledge about the topics being discussed. By analyzing the structure and connections within these networks, researchers can identify dominant themes and understand the contextual use of language within specific communities [64,65]. Figure 4 shows the relationships between the different hashtags created and represents the concepts that are deemed relevant by the community. The size and the color of the vertexes are based on the betweenness centrality of the hashtag (ie, the number of times a node acts as a bridge), whereas the edge size represents the number of users that used both connected hashtags in the same Twitter message. This graph provides valuable information about the public’s interests proposed by the community itself, assisting in the identification of the relationship between the topics that the community is most engaged with and their connection. Figure 4 shows the top comentioned hashtags raised by the community in the social media network. As can be observed, the high frequency of health-related hashtags in the semantic graph suggests that the Twitter community is primarily focused on providing support and information to individuals who may be experiencing symptoms or health issues. This highlights the importance of social media platforms to contribute to public health efforts, such as raising awareness about different health issues and encouraging healthy behaviors.

On the other hand, the hashtag comention analysis also revealed that the community was highly interested in topics such as *news*, *science*, *technology*, *did you know*, and *climate change*. These concepts had the highest betweenness centrality (ie, the number of times a vertex acts as a bridge) and a high degree (ie, a high-frequency mention) and were highly associated with health topics (eg, allergy, hay fever, or asthma). Therefore, this finding suggests that these concepts may play a key role in connecting and bridging different areas of interest and discussion within the community and may be particularly valuable for promoting greater engagement and interaction. In addition, these results indicate that the community was highly interested in staying informed about recent knowledge, scientific discoveries, and technological advancements, as well as advocating for environmental issues such as climate change. These concepts have also been used as keywords in other works; for example, *news* has been indicated as a theme to measure the frequency of tweets discussing *allergy* and *asthma* [63]. Other studies examining health communication during the Ebola outbreak analyzed the topic coverage and sentiment dynamics across 2 different media platforms: Twitter and traditional news publications. The findings suggest that Twitter and news media each presented distinct perspectives on the issue [66]. Another concept such as *technology* was used in a study that developed an app called Allergy Diary, in which the authors concluded that mobile technology is likely to become an important tool to better understand and manage allergenic rhinitis and asthma [67].

**Figure 5:**
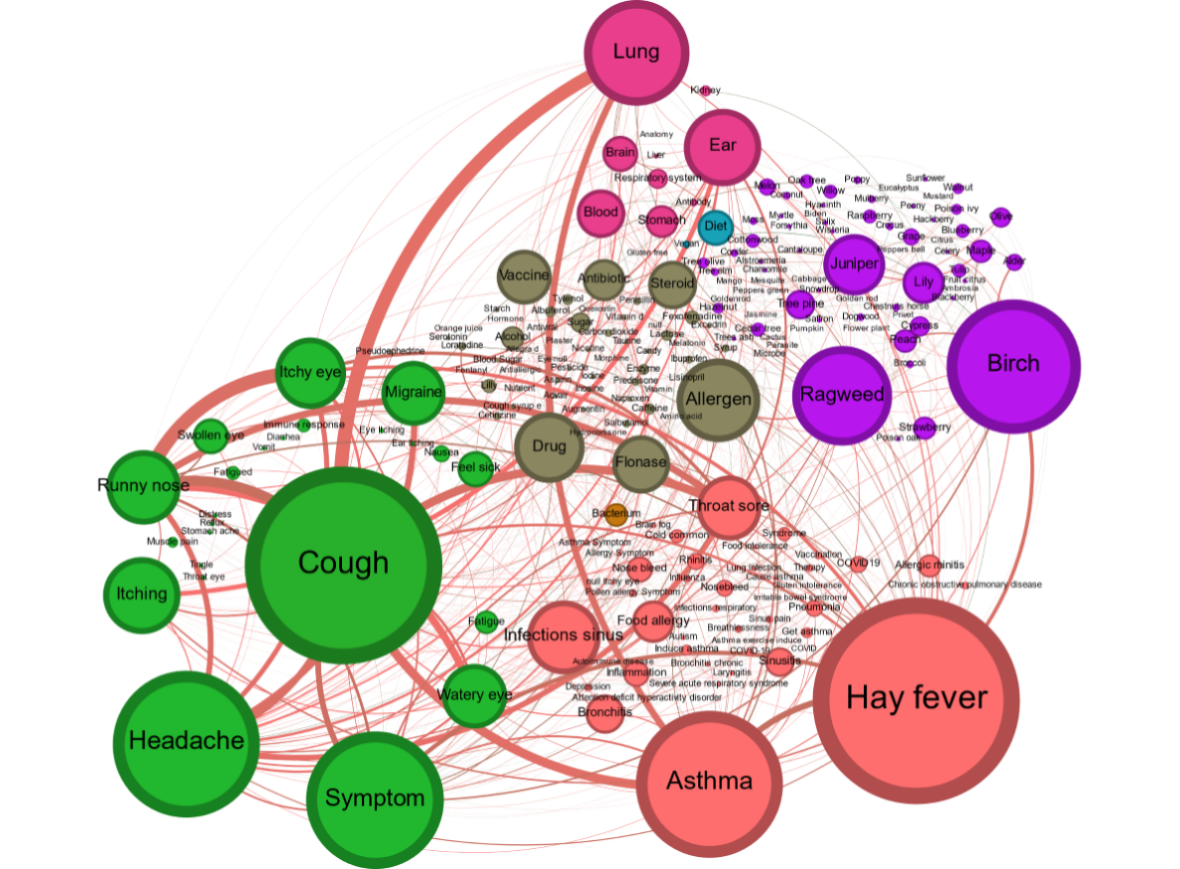
Top comentioned health-related topics identified in first-person messages. Knowledge graph representing the comentioned health-related topics identified from December 2006 to January 2022 in pollen-related discussions.

On the other hand, Figure 5 maps out the connections among distinct concepts identified through the NER process, highlighting their co-occurrence within the messages of the community. The vertex color denotes the biomedical domain category annotated (eg, disease), whereas the vertex size is dependent on its degree (ie, the number of annotated interactions). The edge color represents the evaluated sentiment (eg, positive or negative), whereas the width of the edge indicates the number of documents that evidenced the interaction. The semantic graph analysis provides a unique perspective on the health-related topics that are most frequently discussed in first-person messages, including those from patients, and the connections between them to better understand the community’s interests, concerns, and behaviors. This approach offers a more comprehensive and nuanced understanding of the community’s interests and priorities regarding health-related topics to better address the relationships and interactions between different relevant domain topics. These NER processes have been previously applied by other authors in biomedical tasks [68,69] or in the clinical domain [70]. The vertexes in the graph represent identified health-related concepts, whereas the edges denote semantic connections between them, in addition to indicating the predicted sentiment. To enhance the readability and specificity of the knowledge network, generalist terms within the domain, such as *pollen* and *allergy*, were filtered. Therefore, these terms did not contribute significantly to the comprehension of the relationships. By focusing on the most relevant and meaningful terms, the analysis was able to provide a more focused and insightful understanding of relevant health-related concepts.

Regarding the most relevant vertexes, it is evident that *cough*, *asthma*, and *hay fever* were the most prevalent terms, indicating their significance in clinical research. *Cough*, *hay fever*, *headache*, *asthma*, *sore throat*, *eye conditions*, and *sinus infections* were the common health concerns mentioned. Birch and ragweed were the plants or species with the biggest mentions in relation to the identified health-related topics. In terms of graph statistics, the clustering coefficient of the graph is relatively low, indicating that there are not many groups of nodes that are tightly connected to each other. This means that the community had a broad discussion about medical terms without focusing on a specific relationship. However, some nodes, such as *sore throat* and *sinus infection*, have a relatively high clustering coefficient, indicating that they are part of highly connected subnetworks (ie, most commonly mentioned symptoms concerning different health-related topics). On the other hand, some notable nodes, including *cough*, *hay fever*, and *headache*, have the lowest average shortest path lengths, meaning that they are connected to many other nodes and are highly central in the graph. This suggests that these symptoms were more likely to be mentioned in tweets related to pollen allergies and first-person experiences. Finally, a comparison between the hashtag and health-related mention graphs revealed that, while health-related terms associated with hashtags primarily centered on respiratory issues and eye-nose conditions (eg, *cough*, *asthma*, or *itchy eyes*), there was a notable lack of mentions related to symptoms such as *headaches*, *migraines*, or *fatigue* mentioned in the first-person term analysis graph. Such findings suggest a possible lack of community support or awareness concerning these conditions.

An analysis of the sentiment expressed in the knowledge graph revealed a predominance of negative sentiment. These data were aligned with those of another study focusing on tweets related to both *asthma* and *allergic rhinitis* that found that the average sentiment scores were predominantly negative [63].

The most frequently mentioned medical conditions were *watery eyes*, *runny nose*, *hay fever*, *headache*, and *migraine*, along with *cough* and *asthma*. However, there is also evidence of some positive sentiment relationships in the dataset, with a concentration of positive mentions around certain medications, such as *Flonase*, *Tylenol*, and *Cetirizine*, as well as improvements in symptoms related to hay *fever*, *migraine*, and *cough*.

### Health-Related Knowledge Analysis

**Figure 6:**
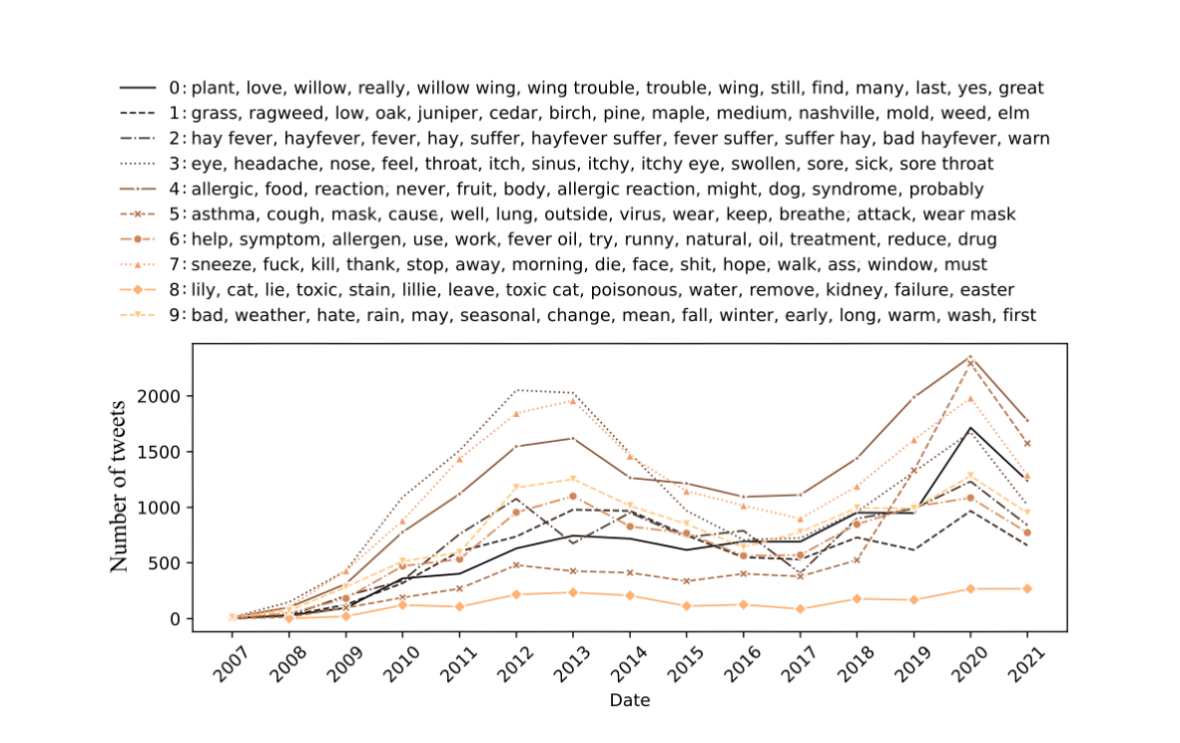
Evolution of Twitter conversations related to “pollen” within the recognized topic, based on Twitter messages containing at least one medical term, from December 2006 to January 2022.

To gain a deeper understanding of the medical knowledge contained in the messages and identify concepts and relationships that were not previously identified by the ontologies and NER techniques, an unsupervised analysis was conducted on all relevant messages that contained at least one medical term, identified through the proposed annotation workflow. The objective of this analysis was to identify any previously undiscovered correlations between the medical concepts mentioned in the messages, thus providing a more comprehensive understanding of the relationships between them. Table 3 represents the most discussed topics measured by their TF-IDF, and Figure 6 shows their evolution over time.

The unsupervised analysis of health-related messages reveals a wide-ranging discourse, from environmental appreciation and specific allergens (topic 0) to individual experiences with hay fever and associated symptoms (topics 2 and 3). Topic 1 revealed a comprehensive dialogue around distinct types of pollen and other allergens that can affect air quality and health. Topics such as topic 4 (allergic reactions and food) and topic 5 (asthma and respiratory issues) extended the conversation to broader allergy and respiratory conditions, suggesting a crossover between pollen allergies and other allergic reactions, including dietary concerns and asthma-related challenges. Discussions on treatment strategies (topic 6) highlighted a proactive community seeking both medical and natural remedies. Emotional responses to allergy struggles were vividly expressed (topic 7), whereas unique concerns such as pet and plant toxicity emerged (topic 8). Finally, the impact of weather, seasonal changes, and climatic change on allergies was acknowledged (topic 9), illustrating a comprehensive public understanding of the multifaceted nature of pollen allergies.

**Table 3:** Top 6 main topic clusters showing the different concepts discussed together in messages with identified health-related named entity recognition annotations from December 2006 to January 2022.

		TF-IDF^a^
**Topic 0**		
	Plant	0.18
	Love	0.12
	Willow	0.12
	Really	0.10
	Willow wing	0.10
	Wing trouble	0.10
	Trouble	0.10
**Topic 1**		
	Grass	0.34
	Ragweed	0.22
	Low	0.21
	Oak	0.21
	Juniper	0.19
	Cedar	0.18
	Birch	0.17
**Topic 2**		
	Hay fever	0.46
	Hayfever	0.45
	Fever	0.42
	Hay	0.42
	Suffer	0.18
	Hayfever suffer	0.07
	Fever suffer	0.06
**Topic 3**		
	Eye	0.43
	Headache	0.25
	Nose	0.22
	Feel	0.19
	Throat	0.14
	Itch	0.13
	Sinus	0.13
**Topic 4**		
	Allergic	0.56
	Food	0.09
	Reaction	0.07
	Never	0.06
	Fruit	0.06
	Body	0.05
	Allergic reaction	0.04
**Topic 5**		
	Asthma	0.35
	Cough	0.17
	Mask	0.14
	Cause	0.13
	Well	0.11
	Lung	0.11
	Outside	0.10
**Topic 6**		
	Help	0.18
	Symptom	0.13
	Allergen	0.11
	Use	0.10
	Work	0.10
	Fever oil	0.09
	Try	0.08
**Topic 7**		
	Sneeze	0.21
	Fuck	0.10
	Kill	0.08
	Thank	0.08
	Stop	0.07
	Away	0.07
	Morning	0.06

^a^TF-IDF: term frequency–inverse document frequency.

### In-Depth Analysis of Previously Discovered Topics

#### Overview

In terms of health-related analysis, there were different key concepts discovered in previous sections (Figures 3, 4, and 5) that require further analysis to gain a deeper understanding of the correlation between these terms and the pollen domain. In this line, an expert in the field carefully selected these topics, revising the previous sections and focusing on areas that were not directly associated with respiratory issues or topics that demanded more detailed examination. For instance, pine pollen grains were traditionally considered a nonallergenic pollen, and their relevance in this work deserves further attention [71]. This expert-driven selection ensured that the analysis covered both expected and less obvious aspects related to pollen, providing a comprehensive view of the topic’s impact on public health discussions. To accomplish this objective, a compilation of all relevant messages that refer to the specific terms outlined in the following sections was gathered and analyzed.

#### Headache and Migraine

While respiratory-related illnesses dominate health discussions, headaches and migraines were among the most commonly reported symptoms in the evaluated dataset. Therefore, given the significant impact that headaches and migraines can have on individuals’ daily lives and overall well-being, further research in this area could provide new insights into strategies for prevention, management, and treatment, potentially enhancing the quality of life. Textbox 2 represents the most common unsupervised concepts related to this topic.

**Most common words and bigrams related to the headache and migraine discussions based on their term frequency–inverse document frequency.**

**Top 10 relevant words**

- Giving
- Sinus
- Hate
- Bad
- Nose
- Worst
- Outside
- Sneezing
- Migraines
- Away

**Top 10 relevant bigrams**

- Eye-nose
- Migraine-sinus
- Sinus-wake
- Hate-sinus
- Rain-wash
- Nose-throat
- Sinus-thank
- Eye-sinus
- Morning-wake
- Cause-sinus

The comentioned words in the headache and migraine discussions reveal a clear comorbidity of the reported illness with sinus and sneezing, suggesting that these symptoms may be related to or exacerbate the patient’s condition. These findings are comparable with those of another study that investigated the relationship between allergic rhinitis and migraines, implying a probable association [72]. Nevertheless, more recent studies suggest that allergens and headaches or migraines may not be related [73], emphasizing the need for greater research to fully understand any potential connections. In addition, there appeared to be a seasonal pattern in the reported symptoms, with a higher frequency of mentions referring to a specific time of the day. Notably, the data suggest that symptoms may be more concentrated in the morning, as evidenced by the prevalence of terms such as *morning*, *wake*, and *sleep*. This result aligns with the observations of a study conducted in the northeastern United States between May 2020 and June 2020, which identified that terms such as *morning* and *sleep* were predominantly used to categorize clusters of conversations using hierarchical clustering of subgraphs [73].

**Figure 7:**
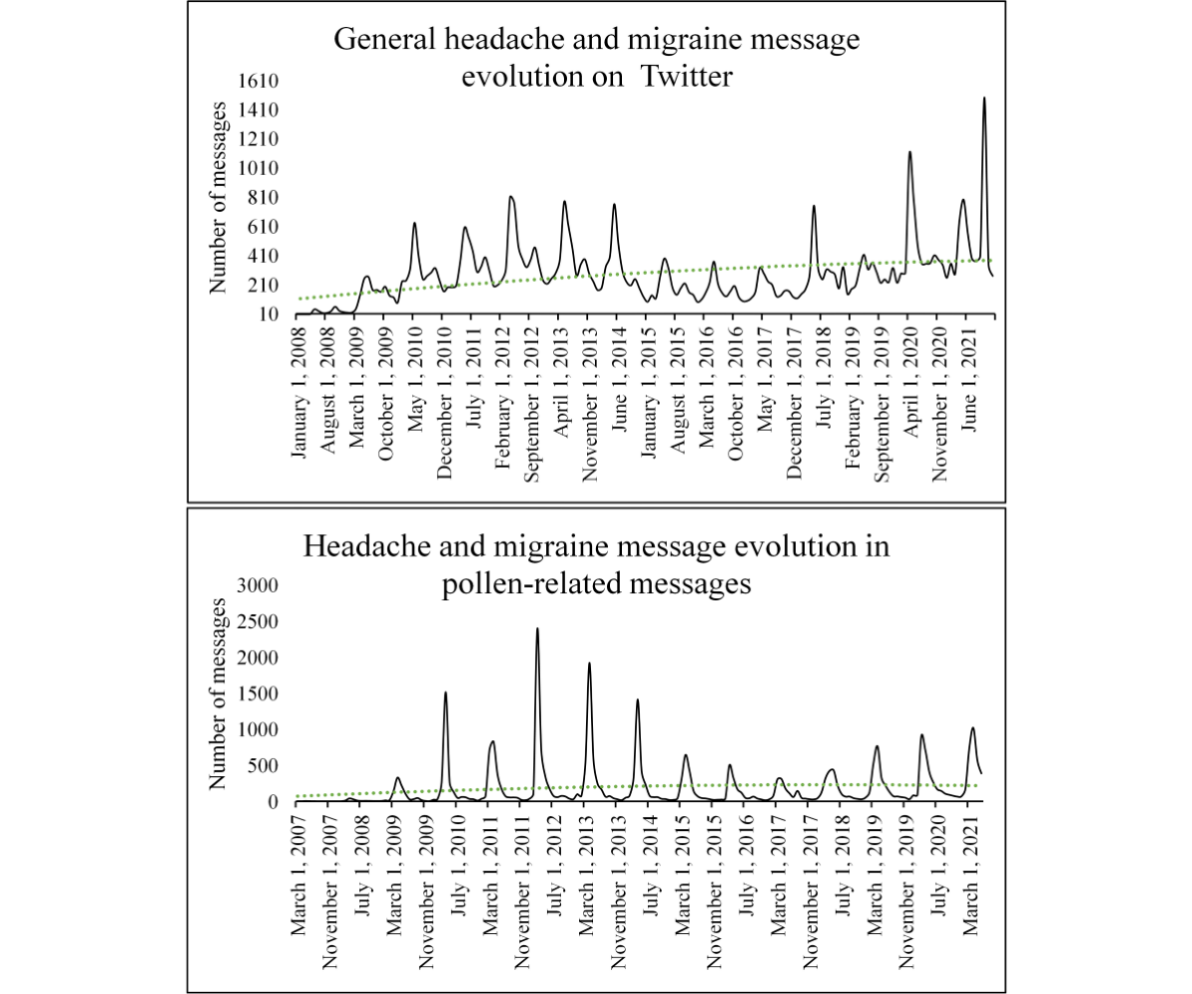
Evolution of headache and migraine mentions on Twitter (top) and in pollen-related messages (bottom).

Figure 7 illustrates the changes in the frequency of mentions of migraines and headaches over time, with a focus on their stationarity. The top chart presents the number of messages mentioning headache and migraine obtained from the Twitter application programming interface. The bottom chart presents the number of messages mentioning both symptoms and pollen. The green line represents a polynomic tendency line of grade 2. In this sense, the data show a distinct pattern of stationarity between March and September, with the largest number of mentions (considering the total number of messages) recorded in 2012 and 2013. However, in terms of discussion volume, the last years showed that there was a growing tendency to discuss these conditions, indicating a possible increasing significance and impact on people’s lives. Overall, these findings suggest the need for further research to better understand the comorbidity of headaches and migraines with sinus and sneezing symptoms, as well as the potential influence of time of day on the occurrence of these conditions.

#### Pine

According to the findings, the plants that were mentioned most frequently in the community were ragweed, oak, cedar, pine, and birch. In this sense, one of the most mentioned plants was pine even though allergies to pine pollen are extremely rare [74]. To further explore the public discourse regarding pine, a topic analysis was conducted to analyze the public discussion along this line. Textbox 3 presents the most mentioned topics concerning pine pollen. Notably, a considerable number of mentions on this topic revolved around the belief that natural compounds contained in pollen have the potential to boost testosterone levels and, as a result, enhance physical performance. This concept sparked debates regarding the use of pine pollen as a natural supplement aimed at boosting testosterone-related attributes.

**Most common words and bigrams related to pine based on their term frequency–inverse document frequency.**

**Top 10 relevant words**

- Hormones
- Testosterone
- Pine
- Natural
- Levels
- Bee
- Sex
- Boosting
- Allergies
- Balance
- Increase

**Top 10 relevant bigrams**

- Feel-hormone
- Hormone-mix
- Count-hormone
- Hormone-need
- Bee-hormone
- Cry-hormone
- Hormone-stress
- Hormone-woman
- Hormone-plant
- Headache-hormone
- Blame-hormone

#### Asthma

To enhance our comprehension of the primary topics connected to asthma and associated concerns, Textbox 4 illustrates the terms most frequently mentioned alongside this health condition. The analysis identified *pregnancy*, *mask*, and *climate change* as the most prevalent subjects in asthma-related discussions. This finding aligns closely with the observations presented in Figure 6 regarding female individuals. Specifically, the prominent frequency of discussions related to pregnancy and asthma within the community indicates a strong interest among pregnant individuals in obtaining information and support for managing asthma during pregnancy, with the goal of minimizing potential risks to their unborn children. This underlines the critical need for making reliable, easily accessible information available to those living with asthma and underscores the necessity for heightened awareness and education on managing asthma during pregnancy among health care providers and the wider community.

On the other hand, the discussion of climate change highlights the increasing concern about the impact of environmental factors on respiratory health. Climate change can exacerbate asthma by increasing air pollution, triggering more frequent and severe allergic reactions and leading to extreme weather events that can cause respiratory distress and, therefore, increased pollen levels [75]. This finding highlights the heightened sensitivity of the pollen community to the effects of environmental factors on respiratory health.

**Most common words and bigrams related to asthma based on their term frequency–inverse document frequency.**

**Top 10 relevant words**

- Attack
- Triger
- Cause
- Suffer
- Breathe
- Grass
- Mask
- Help
- Feel
- Fever
- Weather

**Top 10 relevant bigrams**

- Fever-hay
- Change-climate
- Mask-wear
- Change-link
- Increase-risk
- Change-weather
- Kid-trigger
- Exposure-pregnancy
- Attack-trigger
- Child-risk
- Fever-hay

#### Tired, Fatigue, Kill, and Sleep

The analysis of the terms *tired*, *fatigue*, and *sleep* in relation to the main topic of pollen allergies revealed interesting insights (Textbox 5). These symptoms were highly associated with watery eyes and runny nose, indicating that this symptoms have a significant impact on sleep quality, resulting in tiredness and fatigue during the day. These results are in accordance with the findings of a study conducted in South Korea, in which the authors pointed out that individuals with allergic rhinitis had significantly greater sleep disorders, fatigue, and depressive symptoms than those without allergic rhinitis, so allergic rhinitis was an important factor influencing sleep disorders and fatigue [76]. Moreover, the analysis carried out highlight a possible association between pollen-related health conditions and work performance, suggesting that pollen allergies can negatively impact work productivity. However, a recent meta-study indicates that the relationship between allergic symptoms and sleep problems or daytime fatigue ranges from low to very low, suggesting caution when interpreting these results [77]. This implies that the findings of this study may be more reflective of patient perceptions than of confirmed interrelated symptoms. In addition, the analysis also indicates that the terms *need-rain* are highly associated with the main topic, suggesting that weather patterns can play a crucial role in mitigating pollen allergy symptoms.

**Most common words and bigrams related to tired, fatigue, and sleep based on their term frequency–inverse document frequency.**

**Top 10 relevant words**

- Eye
- Feel
- Rain
- Fuck
- Hate
- Sneeze
- Weather
- Night
- Nose
- Sinus
- Work

**Top 10 relevant bigrams**

- Fever-hay
- Need-rain
- Rain-wash
- Open-window
- Eye-nose
- Eye-itch
- Mask-weather
- Burn-eye
- Cut-grass
- Love-weather
- Feel-head

#### Sinus Conditions

To gain a better understanding of the principal key topics related to sinus conditions, Textbox 6 represents the most comentioned terms related to this health condition. Regarding the mentioned words, headache and infection were some of the most mentioned cosymptomatology. Furthermore, the significance of discussions centered around mornings and the related health conditions, such as ear infections, should also be emphasized.

**Most common words and bigrams related to sinus based on their term frequency–inverse document frequency.**

**Top 10 relevant words**

- Fuck
- Headache
- Infection
- Hate
- Feel
- Kill
- Hate
- Snort
- Eye
- Weather
- Rain

**Top 10 relevant bigrams**

- Fever-hay
- Rain-wash
- Mask-wear
- Eye-nose
- Change-weather
- Ass-kick
- Ear-infection
- Need-rain
- Feel-hope
- Feel-head
- Kill-try

### Shared Resource Analysis

An analysis of the most shared resources (ie, URLs), grouped by topic using the unsupervised workflow, was carried out to reveal valuable insights into the most relevant and popular shared resources on the Twitter platform. Web-based data were scraped and grouped by topic considering their website descriptions, titles, and contents. It should be noted that not all websites could be obtained as some of the resources may no longer exist at present. This information can be used to better understand the needs and interests of the community and tailor communication and content strategies accordingly. In short, analyzing shared URLs is a powerful tool for gaining knowledge about the most important and interesting topics in the community that endure over time. In addition, by analyzing popular websites, it is possible to identify key influencers and thought leaders across various domains, facilitating targeted networking and outreach strategies. Insights derived from these patterns are invaluable for understanding community interests and trends, thereby enhancing strategic decision-making and communication initiatives.

**Figure 8:**
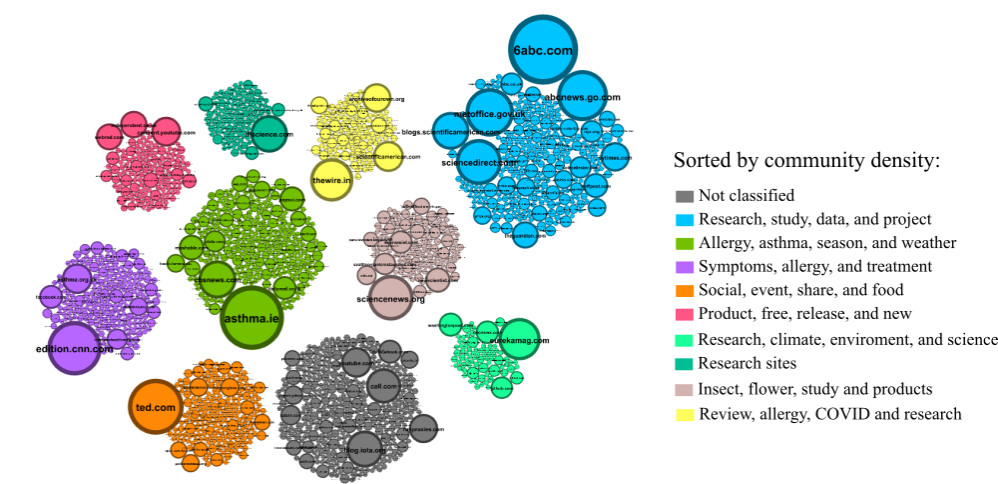
Top shared resources by the community. This figure shows the most shared URLs on Twitter grouped by topic using website embeddings for clustering.

Figure 8 shows the top shared resources by the community as well as unsupervised classified topics sorted by their share density (ie, the number of users mentioning the URL). Each bubble represents a domain, with colors indicating the topic and bubble sizes reflecting popularity based on user mentions. Major shared topics include news, research, pollen studies, symptoms, weather, and treatments, highlighting community interests in health and medical updates. As can be observed, the most valuable resources for the community were primarily related to news portals, research information, and new related pollen research projects. In addition, resources related to symptoms, weather, and treatments were highly valued by the community. This suggests that the community is highly interested in staying informed about the latest research and developments in the field of health and medicine. Furthermore, the significance of resources related to symptoms, weather, and treatments suggests that the community is striving to gain a better understanding of their health conditions and find effective ways to manage their symptoms, as well as be prepared for weather and climate conditions. Overall, these findings provide insights into the information needs of the community and can be used to inform the development of targeted resources and interventions to better support and empower individuals with health-related concerns.

## Discussion

### Principal Findings

This study used different NLP and text mining techniques to analyze 4.6 million tweets (from December 2006 to January 2022) about pollen allergy symptomatology and its influence on patients with allergy. The study found 1.39 million relevant tweets, with most messages posted between March and August every year. In terms of message evolution, there was a significant rise in patient tweets concerning pollen-related health issues. Unsupervised analysis revealed a wide range of subjects, including environmental variables, personal hay fever experiences, allergic responses, asthma, and medicine, illustrating the complexities of pollen allergies. The presence of emphatic language in tweets reflects the frustration and discomfort experienced by those who have allergies. The association of pollen with weather-related terms such as *rain*, *wind*, and *mold* suggests that public awareness and concerns about pollen allergies are intricately linked to environmental conditions. This finding is consistent with previous studies that measured the frequency of weather-related words in tweets discussing *allergy*, *asthma*, and *rhinitis*. Furthermore, associations were found between medical concepts such as the comorbidity of headaches and migraines with nasal symptoms and sneezing, as well as a relevant debate concerning sleep problems and fatigue-related symptoms associated with pollen allergy symptoms. On the other hand, misinformation was found about the use of pine pollen as a stimulant for the generation of testosterone.

### Strengths and Limitations

While this study contributes to the understanding of pollen-related discourse on social media platforms, several limitations and strengths should be noted. First, the data used in this analysis were sourced exclusively from a publicly available social media platform, which may not fully capture the breadth and depth of discussions on the topic. In addition, inherent biases within social media data, such as fluctuations in user engagement and platform popularity, may influence the findings. However, the use of publicly available data on Twitter allows for a large and diverse study, providing a broad overview of public sentiment and awareness. In this way, Twitter has become a highly valued source of knowledge in the scientific community [78]. A significant strength of this work lies in the use of public and distended Twitter data. The voluntary nature of the data used in this work also means that it reflects genuine and spontaneous patient experiences and concerns, making it a rich source of information.

On the other hand, the generalizability of the findings may be constrained by the language and geographical biases inherent in social media data and introduces the possibility of an impact on the interpretation of social media discourse on pollen. However, this inherent bias is a widespread challenge that highlights the multilayered complexities involved in interpreting social media data [79,80]. Conversely, the primary advantage of this work lies in the extensive reach of social media and data volume, allowing for the collection and analysis of a vast amount of social information from a diverse and widespread user base. This comprehensive dataset provides a more globalized perspective, enabling the identification of aggregate patterns and trends that might be overlooked in studies with smaller and regionalized sample sizes. This inclusivity enhances the reliability and relevance of the findings, making them more applicable to different demographic and geographic contexts.

Finally, the similarity between allergic symptoms and those of some viral infections introduces an inherent bias in analyzing social media discussions about pollen. Despite focusing the data query specifically on messages that contained the word *pollen*, reducing the likelihood of capturing discussions related to unrelated viral illnesses or common colds, it is not possible to eliminate this bias. The difficulty that individuals face in distinguishing between allergic and viral symptoms, which is well documented in the literature, must be acknowledged when interpreting the findings of this study [81]. Nevertheless, a significant strength of this work lies in its ability to focus the results only on those individuals who offer information about their health or perceptions concerning the search query of this work (*pollen*), which may minimize the incorporation of discussions that deal exclusively with viral infections. In addition, the use of sophisticated analytical tools in this work, such as modern LLMs, enhances the reliability of the analysis and emphasizes the robustness of our methodology. These models enable precise differentiation of the discussed medical concepts based on their context. LLMs automatically distinguish between discussions of allergic and viral symptoms in contexts where these symptoms are explicitly mentioned.

### Comparison With Prior Work

Health-related social network data have been used for large-scale analysis of issues related to *pollen* and other medical ailments, known as *infovigilance* [82]. Nonetheless, this work conducts a more globalized analysis of pollen and pollen allergy than other regional research, concentrating the analysis on more aggregated data rather than on targeted discussions [83-85]. This comprehensive analysis facilitates the identification of common themes and the discovery of patterns among patients from various regions worldwide. The findings of this study align with those of earlier studies showing the seasonality of social media activity related to health and the influence of environmental factors on respiratory disorders. Similar to earlier findings, the frequent correlation between tweets about pollen allergies and phrases such as *weather*, *wind*, *rain*, and *climate change* demonstrates that public concerns are related to climatic changes [83]. On the other hand, in 2020, the evaluated data revealed a peak in *pollen* tweets, which coincided with a surge in Google Trends related to media coverage of asthma [86]. In terms of seasonality, our data on pollen discussions fluctuated over the years, with a visible decrease between 2013 and 2018. This information does not fully align with reports showing a rising prevalence of allergies in recent years [87] and those indicating that an increase in carbon dioxide concentrations and atmospheric temperatures elevate pollen levels, causing high pollen counts to start earlier and last longer [88]. However, individual perceptions of allergic symptoms may be influenced by emotional and external factors, such as community attitudes and general perceptions. Therefore, some studies suggest that the impact of allergies, personal experiences, and perceptions can be significantly affected by emotional states [89,90].

### Conclusions

The primary value of tweets is the insights that they provide into human behavior and thought processes; people share their genuine, spontaneous experiences related to pollen, providing information that would otherwise be difficult to obtain. In this sense, this study provides a detailed understanding from various perspectives of how pollen allergies are discussed on Twitter. In addition, it explores how incorporating modern LLMs can enhance the analysis and understanding of the public discussion. These advanced models can analyze vast amounts of text data, identifying patterns and nuances that traditional methods might overlook. The scientific understanding of pollen and allergies can greatly benefit from alternative data sources, including social media, to improve and validate related scientific knowledge. By analyzing the causal relationships among pollen counts, tweet numbers, and semantic patterns related to pollen-related health issues, valuable insights can be gained into how patients interact with pollen information. These insights can assist health stakeholders, organizations, and governments in monitoring public engagement, guiding decision-making, and designing targeted communication strategies. This approach may prove valuable in adopting preventive measures to mitigate the impact of pollen allergies on sensitive patients and improving access to health information. Leveraging social media data for public health surveillance and intervention has the potential to advance the development of new treatments and improve the overall management of pollen allergies. This study is expected to help public health officials, researchers, and medical professionals better understand the pollen-related community and use Twitter more efficiently for various objectives, such as tracking symptoms, disseminating information, and adopting preventive measures.

## Abbreviations

ABNER: A Biomedical Named Entity Recognizer
BERT: Bidirectional Encoder Representations From Transformers
DNORM: disease name normalization
KNN: k-nearest neighbor
LLM: large language model
NER: named entity recognition
NLP: natural language processing
OSCAR4: Open Source Chemistry Analysis Routines
PCA: principal component analysis
TF-IDF: term frequency–inverse document frequency
